# Association of Race and Poverty Status With DNA Methylation–Based Age

**DOI:** 10.1001/jamanetworkopen.2023.6340

**Published:** 2023-04-07

**Authors:** Botong Shen, Nicolle A. Mode, Nicole Noren Hooten, Natasha L. Pacheco, Ngozi Ezike, Alan B. Zonderman, Michele K. Evans

**Affiliations:** 1 Laboratory of Epidemiology and Population Sciences, National Institute on Aging, National Institutes of Health, Baltimore, Maryland

## Abstract

**Importance:**

The Dunedin Pace of Aging Calculated From the Epigenome (DunedinPACE) measure is a newly constructed DNA methylation (DNAm) biomarker associated with morbidity, mortality, and adverse childhood experiences in several cohorts with European ancestry. However, there are few studies of the DunedinPACE measure among socioeconomically and racially diverse cohorts with longitudinal assessments.

**Objective:**

To investigate the association of race and poverty status with DunedinPACE scores in a socioeconomically diverse middle-aged cohort of African American and White participants.

**Design, Setting, and Participants:**

This longitudinal cohort study used data from the Healthy Aging in Neighborhoods of Diversity Across the Life Span (HANDLS) study. HANDLS is a population-based study of socioeconomically diverse African American and White adults aged 30 to 64 years at baseline in Baltimore, Maryland, with follow-up approximately every 5 years. The current study was restricted to 470 participants with blood samples at 2 time points: August 14, 2004, to June 22, 2009 (visit 1), and June 23, 2009, to September 12, 2017 (visit 2). Genome-wide DNAm was assessed at visit 1 (chronological age, 30-64 years) and visit 2. Data were analyzed from March 18, 2022, to February 9, 2023.

**Main Outcomes and Measures:**

DunedinPACE scores were estimated for each participant at 2 visits. DunedinPACE scores are values scaled to a mean of 1, interpretable with reference to a rate of 1 year of biological aging per 1 year of chronological aging. Linear mixed-model regression analysis was used to examine the trajectories of DunedinPACE scores by chronological age, race, sex, and poverty status.

**Results:**

Among 470 participants, the mean (SD) chronological age at visit 1 was 48.7 (8.7) years. Participants were balanced by sex (238 [50.6%] were men and 232 [49.4%] were women), race (237 [50.4%] African American and 233 [49.6%] White), and poverty status (236 [50.2%] living below poverty level and 234 [49.8%] living above poverty level). The mean (SD) time between visits was 5.1 (1.5) years. Overall, the mean (SD) DunedinPACE score was 1.07 (0.14), representing a 7% faster pace of biological aging than chronological aging. Linear mixed-effects regression analysis revealed an association between the 2-way interaction between race and poverty status (White race and household income below poverty level: β = 0.0665; 95% CI, 0.0298-0.1031; *P* < .001) and significantly higher DunedinPACE scores and an association between quadratic age (age squared: β = −0.0113; 95% CI, −0.0212 to −0.0013; *P* = .03) and significantly higher DunedinPACE scores.

**Conclusions and Relevance:**

In this cohort study, household income below poverty level and African American race were associated with higher DunedinPACE scores. These findings suggest that the DunedinPACE biomarker varies with race and poverty status as adverse social determinants of health. Consequently, measures of accelerated aging should be based on representative samples.

## Key Points

**Question:**

As a biomarker of biological aging, does DNA methylation (DNAm) adequately assess the rate of change in aging over time among socioeconomically diverse middle-aged African American and White adults?

**Findings:**

In this cohort study of 470 socioeconomically diverse middle-aged African American and White adults, household income below poverty level and African American race were associated with a higher DNAm-based pace of aging.

**Meaning:**

These findings suggest that the DNAm-based pace of aging may provide insight into how social constructs such as race play a role in healthy aging.

## Teaser

This cohort study uses data from participants in the Healthy Aging in Neighborhoods of Diversity Across the Life Span study to assess the association of race and poverty status with the DNA methylation–based pace of aging among socioeconomically diverse middle-aged African American and White adults.

## Introduction

Aging is the accumulation of biological and physiological changes associated with dysfunction over time. Chronological age measures time passed since birth. The concept of biological aging represents changes over time in pathophysiological and organismal function.^1,2^ Biological aging is a multicomponent process including 9 major hallmarks: genomic instability, loss of proteostasis, deregulated nutrient sensing, altered mitochondrial function, cellular senescence, stem cell exhaustion, altered intercellular communication, telomere attrition, and epigenetic alteration.^3^ Biological aging differs among individuals because it is propelled by chronological age, genetics, lifestyle, psychosocial stress, environment, and nutrition among other factors. In populations, chronological age diverges from biological age. Slow agers are those who maintain a healthy phenotype despite advancing age; fast agers develop age-associated disease earlier in their lives. Pinpointing the molecular factors that trigger this disjunction may help identify those at greatest risk of poor health outcomes, discern relevant molecular targets for aging interventions, and provide insight into health disparities.

Understanding the pace of biological aging is the first step toward developing effective clinical interventions to enhance life span and health span. To develop useful biomarkers of biological aging, scientists measure biological age by assessing epigenetic alterations, an aging hallmark. DNA methylation (DNAm) is the most well understood epigenetic alteration in aging and alters gene expression without changing of base sequences. There are specific sites comprising cytosine and guanine separated by a phosphate (CpG) with a methylation state that is altered with age.^4,5,6^

Changing patterns of DNAm at specific CpG sites over the life span determine epigenetic age. Using these DNAm alterations, several groups have developed clocks that use the change of methylation status at specific age-related genomic CpG sites to calculate epigenetic age. Differences between chronological age and epigenetic age define a rate or pace of aging as slow or decelerated (chronological age is greater than epigenetic or biological age), normal (chronological age is equal to epigenetic or biological age), or accelerated (epigenetic or biological age is greater than chronological age).^7^ These DNAm-based clocks have different training phenotypes, including chronological age, life span or mortality, and gestational age.^8^ These measures were devised in the context of differential DNAm at age-related CpG sites^9^; they are proxies that do not directly measure mechanisms of aging processes. The first-generation clocks developed by Horvath and Raj^7^ (hereafter, the Horvath clock) and Hannum et al^10^ (hereafter, the Hannum clock) were trained on chronological age. The second-generation clocks, including the DNAm PhenoAge clock (which measures phenotypic vs chronological age)^11^ and the DNAm GrimAge clock (which measures the association between death and biological age),^12^ were trained on life span for overall mortality. The DNAm PhenoAge clock was developed by regressing a phenotypic measure of risk of death on CpG sites.^11^ The DNAm GrimAge clock was developed by identifying CpG sites associated with plasma biomarkers, then constructing a composite of DNAm-based surrogate biomarkers associated with life span.^12^

Social determinants of health (SDOHs) have implications for aging and longevity. Several studies have tested the associations between various SDOHs and selected first- and second-generation DNAm clocks. These SDOHs include neighborhood deprivation, income, education, childhood adversity, and psychosocial stress. A few cross-sectional studies^13,14,15^ have found associations of socioeconomic status (SES), education, race, and sex with greater age acceleration using some but not all epigenetic clocks. For example, 1 study^16^ including only non-Hispanic White women found that residing in areas of neighborhood deprivation was associated with greater epigenetic age acceleration as defined by the Hannum, PhenoAge, and GrimAge clocks, but not the Horvath clock. Most studies^13,14,15,17^ examining the association between epigenetic age and SES or social adversity used cross-sectional data with equivocal results. One study^17^ used the Horvath clock in the context of low SES and cumulative psychosocial adversity but found no association with longitudinal age acceleration.

Recently, a third wave of DNAm-based biological aging estimators was developed to assess the pace of aging^18^ using within-person longitudinal change to assess decline in multiple physiological measures.^19^ The Dunedin Pace of Aging Methylation (DunedinPoAm) measure was trained on observed changes among participants in the Dunedin Birth Cohort Study (adults born from 1972-1973).^20^ The DunedinPoAm captures the rate of aging using 18 biomarkers and DNAm at CpG sites over 12 years of follow-up. Using this algorithm on several cohorts with different European ancestry, the DunedinPoAm is a reproducible single-point measure of biological aging.^19^ Graff et al^21^ examined the utility of the DunedinPoAm in comparison with first- and second-generation clocks among a cohort of older African American and European American adults. They reported that the GrimAge clock and the DunedinPoAm measure detected faster biological aging among African American participants compared with European American participants, with a mean age of 70 years for both groups.^21^ However, there was no age acceleration observed using the Horvath, Hannum, or PhenoAge clocks among African American participants compared with European American participants.^21^ Subsequently, an improved pace of aging measure was developed with longer longitudinal follow-up of participants in the Dunedin Birth Cohort Study.^22^

The new Dunedin Pace of Aging Calculated From the Epigenome (DunedinPACE) measure was trained on longitudinal clinical data over 20 years using 19 system-integrity biomarkers.^22^ The DNAm data were obtained from a racially homogeneous cohort of White individuals with chronological ages ranging from 26 to 45 years who were from Dunedin, New Zealand. DunedinPACE scores are values scaled to a mean of 1, interpretable with reference to a rate of 1 year of biological aging per 1 year of chronological aging. While most of the first- and second-generation clocks yielded inconsistent results when examining the association between various SDOHs and biological aging, both the DunedinPoAm and DunedinPACE measures identified associations between SDOHs and a significantly faster pace of biological aging.^13,23^ However, these assessments were not longitudinal and the DunedinPACE has not been examined in a racially and ethnically diverse sample. In addition, the studies examined different forms of social adversity.

It is likely that because the various clocks were trained on different characteristics and were developed using homogeneous White samples, the findings provide varying assessments of epigenetic age and the factors associated with it. There is currently no gold standard clock or measure of biological age that pinpoints the social factors or stressors associated with accelerated aging in diverse populations. To further investigate the utility of the DunedinPACE measure in a diverse cohort, we assessed this measure longitudinally among participants in the Healthy Aging in Neighborhoods of Diversity Across the Life Span (HANDLS) study to investigate the association of race and poverty status with biological aging among middle-aged African American and White adults residing in Baltimore, Maryland.

## Methods

### Study Population

The HANDLS study^24^ is an ongoing prospective population-based longitudinal study with a fixed cohort of community-dwelling African American and White participants. The HANDLS study was initiated in 2004 and designed to disentangle the consequences of race and SES as risk factors for morbidity and mortality.^24^ Participants were recruited from 13 neighborhoods in Baltimore using an area probability sample across sex, race, poverty status, and chronological age. Participants self-identified as either African American or White. Poverty status was defined as higher or lower than 125% of the 2004 US federal poverty guidelines for household income.^25^ Participants had chronological ages ranging from 30 to 64 years at enrollment. Visit 1 data were collected from August 14, 2004, to June 22, 2009; during this period, participants received physical health examinations, medical history inquiries, cognitive testing, and other assessments. Participants were invited for follow-up in-person visits (visit 2: June 23, 2009, to September 12, 2017) using protocols similar to those used in visit 1. The 470 participants for the current cohort study were selected across a factorial design of sex, race, and poverty status from a group of participants with at least 2 blood samples from visit 1 (2004-2009) and visit 2 (2009-2017)^26^ (Table 1). There was a mean (SD) of 5.1 (1.5) years between visit 1 and visit 2. Data were analyzed from March 18, 2022, to February 9, 2023. The HANDLS study protocol was approved by the institutional review board of the National Institutes of Health. All participants provided written informed consent, including permission to use and publish their data in future studies. This study followed the Strengthening the Reporting of Observational Studies in Epidemiology (STROBE) reporting guideline for cohort studies.

**Table 1.** Participant Demographic Characteristics at Initial Visit by Race and Poverty Status

Characteristic	Participants, No. (%)						
	Total (N = 470)	Race			Poverty status		
		African American (n = 237)	White (n = 233)	*P* value^a^	Above poverty level (n = 234)	Below poverty level (n = 236)	*P* value^a^
Sex							
Female	232 (49.4)	117 (49.4)	115 (49.4)	>.99	117 (50.0)	115 (48.7)	>.99
Male	238 (50.6)	120 (50.6)	118 (50.6)		117 (50.0)	121 (51.3)	
Race							
African American	237 (50.4)	NA	NA	NA	120 (51.3)	117 (49.6)	>.99
White	233 (49.6)	NA	NA		114 (48.7)	119 (50.4)	
Poverty status							
Above poverty level	234 (49.8)	120 (50.6)	114 (48.9)	.78	NA	NA	NA
Below poverty level	236 (50.2)	117 (49.4)	119 (51.1)		NA	NA	
Chronological age, mean (SD), y	48.7 (8.7)	48.4 (8.8)	49.0 (8.7)	.58	48.7 (8.7)	48.7 (8.8)	.53
DunedinPACE score, mean (SD)	1.07 (0.14)	1.09 (0.13)	1.05 (0.15)	.004	1.04 (0.14)	1.10 (0.13)	<.001

Abbreviations: DunedinPACE, Dunedin Pace of Aging Calculated From the Epigenome; NA, not applicable.

^a^ *P* values were derived from a Pearson χ^2^ test of independence (used for sex, race, and poverty status) or a *t* test (used for chronological age and DunedinPACE score).

### DNA Methylation Measures

The DNAm in blood samples was profiled using a methylation array (Human MethylationEPIC BeadChip; Illumina Inc), as described previously.^26^ For each CpG site, the methylation level analyzed was defined as the β value (adjusted for batch effects) and white blood cell proportions. The proportion of each white blood cell type was estimated using the Houseman method,^27^ and β value outliers were excluded. We calculated epigenetic clocks, including the Horvath,^28^ Hannum,^10^ PhenoAge,^11^ and GrimAge^12^ clocks, using the online DNA Methylation Age Calculator developed by the Horvath laboratory.^29^ The residuals of epigenetic age acceleration were obtained by regressing the corresponding DNAm age on chronological age. DunedinPACE scores were calculated from the β values for each participant at 2 time points using the DunedinPACE package for R software developed by Belsky et al.^22^

### Statistical Analysis

Differences among race and poverty status groups were assessed by χ^2^ tests of independence for discrete variables and 2-sided *t* tests for continuous measures. Correlations were tested using Pearson correlation coefficients. Linear mixed-model regression analysis was used to examine the association of chronological age, race, sex, and poverty status with longitudinal measures of DunedinPACE scores. We used backward elimination to select factors for the final model, starting with all possible interactions, and included a quadratic age term (age squared) to test for nonlinear associations with DunedinPACE scores over time. The significance of interactions and the terms were assessed through log likelihood tests, with significance defined as 2-sided *P* < .05. Chronological age was included in the models in decade units centered at age 50 years (50 years was subtracted from age [in years], then divided by 10). All analyses were performed using R software, version 4.2.0 (R Foundation for Statistical Computing).

## Results

### Study Sample Characteristics at Baseline

Overall, there were 470 HANDLS participants included in the current study; of those, 237 (50.4%) were African American, 233 (49.6%) were White, 238 (50.6%) were men, 232 (49.4%) were women, 236 (50.2%) had household incomes below poverty level, and 234 (49.8%) had household incomes above poverty level (Table 1). Participants’ chronological ages ranged from 30.2 years to 65.2 years, with a mean (SD) age of 48.7 (8.7) years at the first visit. There were no significant chronological age differences between participants with household income below vs above poverty level or between African American vs White participants. The mean (SD) DunedinPACE score at initial examination for all participants was 1.07 (0.14), which was significantly greater than 1 (*P* < .001), representing an aging rate that was 7% faster than that of participants in the Dunedin Birth Cohort Study. A notable difference was that individuals with income below poverty level had higher DunedinPACE scores at the initial visit (mean [SD], 1.10 [0.13]) than individuals with income above poverty level (mean [SD], 1.04 [0.14]) (*P* < .001). Chronological age and DunedinPACE scores were positively correlated at the initial visit (Pearson *r* = 0.15; *P* < .001). Of the additional epigenetic clocks used to assess data from the initial visit, we found significant race differences for epigenetic age acceleration using the Hannum clock and significant poverty status differences for epigenetic age acceleration using the PhenoAge and GrimAge clocks (eTable in Supplement 1).

### Longitudinal Analysis of DunedinPACE Scores

**Figure 1.**
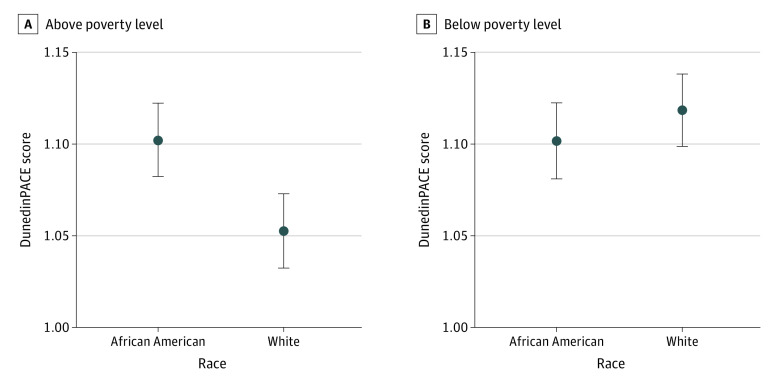
Association of Race and Poverty Status With Dunedin Pace of Aging Calculated From the Epigenome (DunedinPACE) Scores Results of linear mixed-model regression analysis of association of chronological age, sex, race, and poverty status with DunedinPACE scores among 470 participants at 2 time points approximately 5 years apart. Whiskers represent 95% CIs.

Linear mixed-model regression analysis revealed associations of race (White race: β = −0.0497; 95% CI, −0.0755 to −0.0238; *P* < .001), linear age (in decade units: β = 0.0134; 95% CI, 0.0033-0.0234; *P* = .01), quadratic age (age squared: β = −0.0113; 95% CI, −0.0212 to −0.0013; *P* = .03), and the 2-way interaction between race and poverty status (White race and household income below poverty level: β = 0.0665; 95% CI, 0.0298-0.1031; *P* < .001) with significantly higher DunedinPACE scores (Table 2). Overall, White adults living above poverty level had a mean DunedinPACE score of 1.05 (95% CI, 1.03-1.07), which was significantly different from the mean scores of White adults living below poverty level (1.12; 95% CI, 1.10-1.14) and African American adults living either below poverty level (1.10; 95% CI, 1.08-1.12) or above poverty level (1.10; 95% CI, 1.08-1.12; *P* < .001 for interaction) (Figure 1). DunedinPACE scores of the other groups were not significantly different from one another. Sex was not significantly associated with DunedinPACE scores in these analyses.

**Table 2.** Results of Linear Mixed-Model Regression Analysis of Association of Dunedin Pace of Aging Calculated From the Epigenome Score With Race, Poverty Status, and Age at 2 Time Points

Variable	β (SE)	*P* value
Race (White)	−0.0497 (0.0132)	<.001
Poverty status (below poverty level)	−0.0004 (0.0133)	.96
Linear age (in decade units)	0.0134 (0.0051)	.01
Quadratic age (age squared)	−0.0113 (0.0051)	.03
Interaction between race (White) and poverty status (below poverty level)	0.0665 (0.0187)	<.001

**Figure 2.**
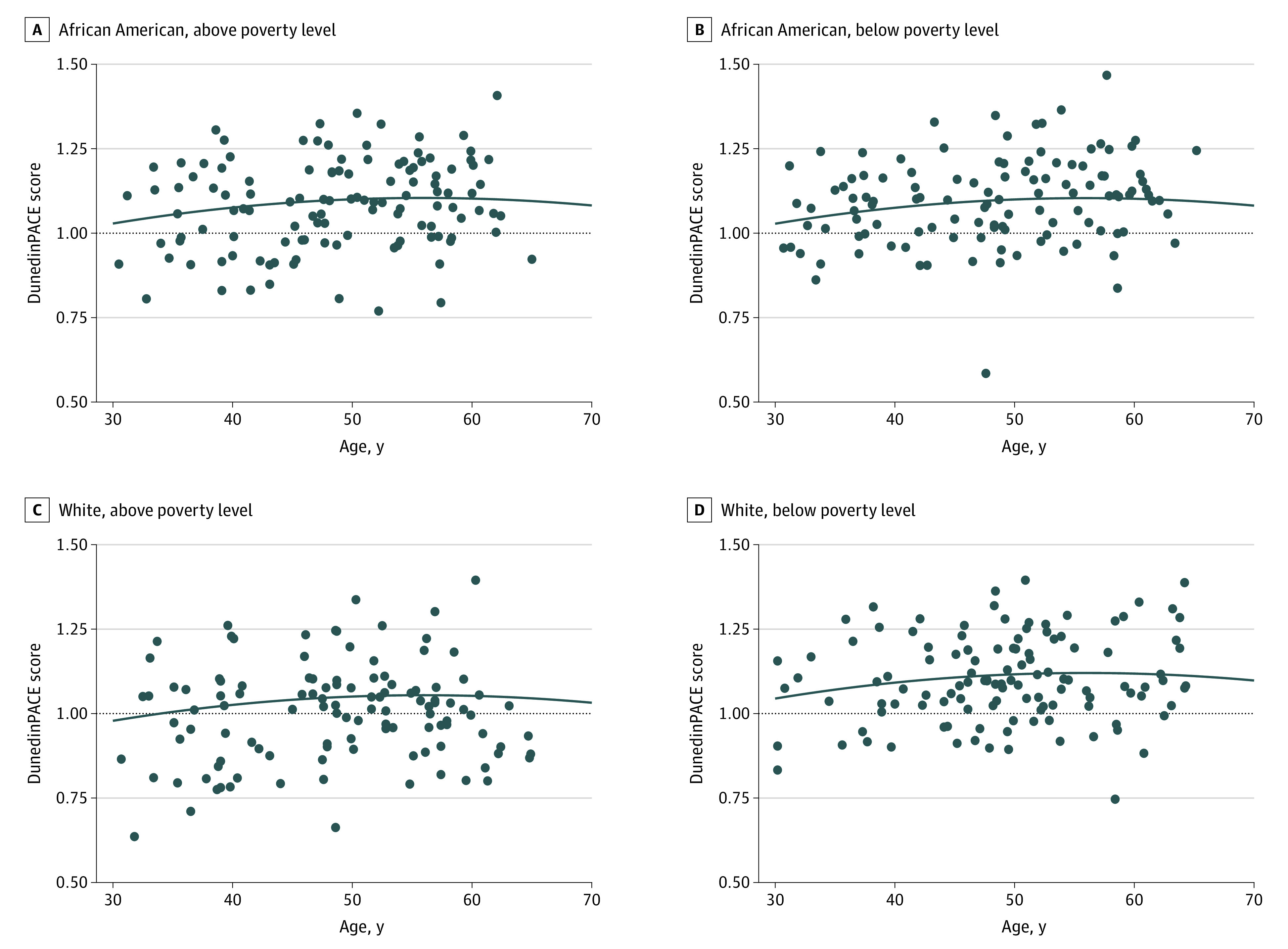
Association of Chronological Age With Dunedin Pace of Aging Calculated From the Epigenome (DunedinPACE) Scores Results of linear mixed-model regression analysis of association of chronological age, sex, race, and poverty status with DunedinPACE scores among 470 participants at 2 time points approximately 5 years apart. The curves show the linear mixed-model regression values for each group across ages 30 to 70 years, and the dots represent data points for each participant’s initial visit (ages 30-64 years). The horizontal dotted line represents a DunedinPACE score of 1.

We also found associations of both linear and quadratic age (which served as measures of time) with significantly higher DunedinPACE scores (Table 2). The quadratic age term yielded an inverted U-shaped association between advancing age and DunedinPACE scores (Figure 2). DunedinPACE scores increased from age 30 years, then decreased after approximately age 55 years. The inverted U-shaped association of chronological age with DunedinPACE scores was independent of race and poverty status and was similar for all participants.

## Discussion

This cohort study found that, overall, the mean DunedinPACE score reflected a pace of aging that was 7% faster than the expectation, defined as the mean physiological decline per year observed in the Dunedin study cohort. Therefore, HANDLS participants in this study were aging 7% more than participants in the Dunedin Birth Cohort Study. In addition, we found that household income below poverty level and African American racial identity were associated with faster biological aging. White participants living above poverty level had a DunedinPACE score near 1, suggesting that biological aging and chronological aging were equivalent. All other groups had higher DunedinPACE scores, which represented faster biological aging than chronological aging. White participants’ DunedinPACE scores differed according to poverty status, with income below poverty level associated with faster biological aging. This was not the case for African American adults in the present cohort. African American participants, regardless of poverty status, had DunedinPACE scores higher than 1. Thus, DunedinPACE scores appeared to exhibit the consequences of adverse exposures for biological aging, particularly underscoring the added liability of race borne by African American participants. The DunedinPACE score cannot distinguish the consequences of poverty status for biological aging in African American individuals because African American racial identity is freighted with disadvantages and psychosocial stressors in US society.

Environmental factors, including psychological stress, alter gene expression and physiological response through changes in the epigenome.^30^ DNAm may be the mediation mechanism for the negative consequences of environmental and psychosocial risk factors. Previous work^26^ using the Hannum and Horvath clocks found that race and sex interacted to produce epigenetic age acceleration. This previous work^26^ did not replicate the association between poverty status and the extant measures of epigenetic age found by other studies that examined groups with low SES,^26,30^ which found evidence of accelerated biological aging. Other studies^15,31,32^ using Horvath and Hannum clocks documented associations of a wide variety of adverse exposures from early life or cumulative exposure with accelerated aging. We did not observe differences in DunedinPACE scores by sex in the present study, which is consistent with work that found no statistically significant differences between men and women.^33^ To our knowledge, the present study is the first to report an inverted U-shaped association between chronological age and DunedinPACE scores. Our ability to identify this association was possible because the DunedinPACE score is a measure of the pace of aging but was not trained on chronological age (like the Hannum and Horvath clocks). Another previous study^34^ reported that DNAm levels at some CpG sites had associations with quadratic age, but more longitudinal studies are needed for verification.

The accelerated pace of biological aging among African American participants overall is not unexpected. However, race is not a biological construct, and SDOHs are an inadequate proxy for social constructs of a myriad of factors including poverty status. The weathering hypothesis posits that health and aging disparities in African American individuals are the product of cumulative socioeconomic disadvantage.^35^ Our findings highlighted the fact that African American individuals experience a disproportionate burden of adverse conditions that is not obviated by living above poverty level, which suggests other SDOHs may be associated with biological aging. These cross-cutting multilevel triggers include both upstream and downstream SDOHs comprising discrimination, education, class inequality, economic opportunity, and housing, among other factors.^36^ A previous study^30^ found contradictory results regarding the association of SDOHs with accelerated epigenetic aging based on Hannum and Horvath clocks. However, in the Family and Community Health Study,^15^ 4 measures of adversity, including education, income, neighborhood disadvantage, and exposure to racial discrimination, significantly estimated accelerated biological aging measured by the GrimAge clock. More recent work^37^ found that neighborhood disadvantage measured by area deprivation index was independently associated with age acceleration using the PhenoAge clock, the GrimAge clock, and the DunedinPoAm measure.

Our finding that living above poverty level was associated with slower biological aging only for White participants validated and highlighted the seminal findings on John Henryism (ie, the high-level effort required to cope with prolonged exposure to stressors such as racial discrimination, resulting in increasing physiological burden among African American individuals) and hypertension among African American adults,^38^ which underscores the race-related burdens imposed by stressful social environments that have adverse consequences for health and wellness. The diminishing returns hypothesis, which has been validated in the literature,^39^ posits that African American adults do not experience the same health benefits of higher SES as White adults in the US. For example, the racial nonequivalence of SES-related health benefits has been documented for college completion, the risk of metabolic syndrome, the costs of social mobility, and biological aging.^40,41^ In fact, the SES gradient associated with biological aging is only partially due to health behaviors, again emphasizing that epigenetic aging is modulated by unmeasured stressful social conditions as well as genetic risk.^13^

The results from previous HANDLS studies^42,43,44,45,46,47^ that focused on poverty status were not completely concordant with the DunedinPACE data. For example, African American men living below poverty level had a significantly increased risk of death compared with those living above poverty level, African American women, and White men and women.^42^ Living below poverty level also has implications for cognition in African American individuals with diabetes,^43^ prevalence of chronic kidney disease among African American but not White participants,^44^ brain volume in African American participants,^45,46^ white matter lesion volume among African American participants,^46^ and differential gene expression among African American participants.^47^ Thus, it appears that the DunedinPACE score correctly assesses the association between poverty status as a measure of social class and biological aging. However, the factors that play a role in biological aging in African American individuals lie at the intersection of race and class. White race and higher household income facilitate opportunities for optimal health outcomes. For African American individuals, race and its concomitant encumbrances quench the benefits of higher household income. The well-known race-related stressors unique to African American individuals include structural racism and the resultant environmental and opportunity deprivations.

Our findings suggest that while poverty status is associated with rates of biological aging, DunedinPACE values are inextricably confounded by social class. In this study, both poverty status and race as a social construct were associated with accelerated biological aging. Although this biomarker is sensitive to adverse life experience,^22^ it cannot disentangle the consequences of race from those of SES, highlighting the fact that race is not a biological construct. If measures of biological aging are proposed as end points in blinded randomized clinical trials of senolytics or other antiaging therapies, then it is important to base these measures on heterogeneous and representative population cohorts in which genetic variation and ancestry are considered. Otherwise, it may be difficult to prove that a clinical benefit for proposed therapies to enhance health span exists for all groups.

### Limitations

This study has some limitations. First, we had a relatively small sample. A larger sample may provide greater power to detect the associations of other covariates, such as sex differences. Second, we used data from 2 time points approximately 5 years apart in this middle-aged sample. This interval may be too short in a relatively young group. Collection of further data from additional time points will be important to test the associations over time and to confirm the curvilinearity of biological age. Third, the effect sizes for our findings were significant but relatively small. Nevertheless, the cumulative consequences suggest a potentially important public health burden as the number of older adults increases over the next several decades.

## Conclusions

The findings of this longitudinal cohort study revealed that race and SES (measured by poverty status) were associated with higher DunedinPACE scores in a middle-aged cohort of African American and White adults. The results suggest that DunedinPACE values reflect the consequences of poverty status on biological aging. Consequently, measures of accelerated aging should be based on representative samples.

## Notes

Supplement 1. **eTable.** Characteristics of Epigenetic Age Acceleration of HANDLS Study Participants at Initial Visit

Supplement 2.
Data Sharing Statement
