# Supplementary material for: Association of Race and Poverty Status With DNA Methylation–Based Age
Source: JAMA Netw Open. 2023 Apr 7;6(4):e236340. doi: 10.1001/jamanetworkopen.2023.6340 (PMC10082406; doi:10.1001/jamanetworkopen.2023.6340)
Supplement: Supplement 1. — eTable. Characteristics of Epigenetic Age Acceleration of HANDLS Study Participants at Initial Visit [file jamanetwopen-e236340-s001.pdf]

# Supplementary Online Content

Shen B, Mode NA, Noren Hooten N, et al. Association of race and poverty status with DNA methylation–based age. *JAMA Netw Open*. 2023;6(4):e236340.
doi:10.1001/jamanetworkopen.2023.6340

**eTable.** Characteristics of Epigenetic Age Acceleration of HANDLS Study Participants at Initial Visit

This supplemental material has been provided by the authors to give readers additional information about their work.

© 2023 Shen B et al. *JAMA Network Open*.

**eTable.** Characteristics of Epigenetic Age Acceleration of HANDLS Study Participants at Initial Visit

| Epigenetic Age Acceleration[a] | Race | | | Poverty Status | | |
|---|---|---|---|---|---|---|
| | African American | White | $P$[b] | Above | Below | $P$[b] |
| **Horvath Accel** (mean ± SD) | -0.22 ± 5.20 | 0.22 ± 4.50 | 0.34 | 0.24 ± 4.74 | -0.24 ± 4.99 | 0.29 |
| **Hannum Accel** (mean ± SD) | -1.58 ± 4.90 | 1.61 ± 3.73 | <0.001 | -0.04 ± 4.80 | 0.05 ± 4.61 | 0.82 |
| **PhenoAge Accel** (mean ± SD) | -0.24 ± 8.00 | 0.25 ± 6.27 | 0.45 | -1.38 ± 6.95 | 1.37 ± 7.18 | <0.001 |
| **GrimAge Accel** (mean ± SD) | -0.51 ± 6.00 | 0.52 ± 6.19 | 0.07 | -1.37 ± 5.83 | 1.36 ± 6.09 | <0.001 |

[a] Age-acceleration residuals of Horvath, Hannum, PhenoAge and GrimAge epigenetic clocks regressing on chronological ages

[b] *P* value from Student's t-test between groups

© 2023 Shen B et al. *JAMA Network Open*.
