# Supplementary material for: Association of Race and Poverty Status With DNA Methylation–Based Age
Source: JAMA Netw Open. 2023 Apr 7;6(4):e236340. doi: 10.1001/jamanetworkopen.2023.6340 (PMC10082406; doi:10.1001/jamanetworkopen.2023.6340)
Supplement: Supplement 2. — Data Sharing Statement [file jamanetwopen-e236340-s002.pdf]

Data Sharing Statement

Shen. Association of Race and Poverty Status With DNA Methylation-Based Age. *JAMA Netw Open*. Published April 07, 2023. doi:10.1001/jamanetworkopen.2023.6340

Data
Data available: Yes
Data types: Other (please specify)
Additional Information: Data can be made available by completing manuscript proposal form found at https://handls.nih.gov/06Coll.htm and returned as an email attachment addressed to: Jennifer H. Norbeck, MSW, CCRC 410-558-8622 norbeckje@mail.nih.gov
How to access data: https://handls.nih.gov/06Coll-dataDoc.htm
When available: With publication

Supporting Documents
Document types: None

Additional Information
Who can access the data: researchers whose proposed use of the data has been approved
Types of analyses: for a specified purpose
Mechanisms of data availability: after approval of a proposal and with a signed data access agreement
